# The role of SMEs in rural development: Access of SMEs to finance as a mediator

**DOI:** 10.1371/journal.pone.0247598

**Published:** 2021-03-08

**Authors:** Faiza Manzoor, Longbao Wei, Noman Sahito

**Affiliations:** 1 Department of Agricultural Economics and Management, School of Public Affairs, Zhejiang University, Hangzhou, China; 2 Department of City & Regional Planning, Mehran University of Engineering & Technology, Jamshoro, Pakistan; International Centre for Integrated Mountain Development (ICIMOD), Kathmandu, Nepal, NEPAL

## Abstract

Small and Medium Enterprises (SMEs) are considered as the fundamental tool for economic growth, nevertheless, they face continuous financing challenges. SMEs are a major source for generating employment, creation of wealth and alleviating poverty from the rural regions in developing countries. Their access to finance is key to the expansion of this sector. The paper aims to discover the intervening role of “access of SMEs to finance” in the link between SME’s evolution and rural development, in the context of Pakistan. In total 338 entrepreneurs operating SMEs in rural areas completed a survey for the study. Through a multi-stage stratified random sampling technique, entrepreneurs were selected from three districts. Confirmatory factor analysis and structural equation modeling were used to test hypotheses. This study shows that SME’s evolution has a positive and optimistic influence on rural development. Further, the study also reveals that on SME’s progress a positive influence happens by the “access of SMEs to finance”. Particularly, the study finds that “access of SMEs to finance” significantly mediated the effect of SME’s evolution on rural development. The findings of this paper hold significant implications for both the research society and loan-issuing institutions and departments.

## Introduction

It is a universal phenomenon that Small and Medium Enterprises (SMEs) are playing an essential and vital role in the nation’s economic and social configuration [1]. "Enterprise is the antithesis of command and control". The worldwide perception of small and micro-businesses or firms has reached noteworthy importance in the economic progress of a nation [2, 3]. Globalization has placed small enterprises unswervingly in the limelight and attention. These are gradually and progressively the main strength for national economic development. All over the world, the entrepreneurs who operate them are getting thoughtful attention from planners, economists, governments, and multilateral agencies [4]. In developing countries, this sector is beneficial in the development of rural regions, and have significance in poverty alleviation [5].

Pakistan is one of the developing countries and the sixth most populated country on earth with over 212 million people. Recently, this country has faced a range of challenges, such as high unemployment, extreme poverty, and slow growth in the development process [6]. Therefore it is quite challenging to ensure employment to this huge population by government or public institution only. So private sector involvement in this development process is equally important [7]. The private sector mainly consists of micro, small and medium enterprises that produce a large share of employment and income opportunities. SME development could be an emerging force of entrepreneurship development, employment generation, and poverty alleviation for any least developed country [8]. Nevertheless, their development potentials remain untouched, as firms that operate in isolation are locked into competitive production patterns and unable to approach dynamic business partners that could bring in new expertise and know-how. It is the role of the government to facilitate private sector development as well as create and maintain the competitive private sectors and contribute to poverty reduction by building sustainable linkage among small-size enterprises, their large scale business partners, and support institutions [9]. Hence, the government has to develop the SME sector, which has the potential to generate employment, and simultaneously they (government) have to certify that the youth are provided excellent education and quality training for operating a more productive SME sector for the economy [10].

The greater growth potential resides in the emerging high-tech industries but is also present in the conventional small business sector’s labor-intensive industries and even in the services that sustain it [11]. "Young people in the future are more likely to end (therefore) should be working in organizations closer to the entrepreneurial model" [12].

This sector is a central pillar for creating job opportunities, the elevation of invention, overcoming struggle, and a vigorous economy [13]. Many developing countries are reaping the benefits of export from SMEs [14]. But they are not extending these benefits to a larger extent. For instance, rural people of Pakistan have a low-level of living standards and development. For developing nations like Pakistan, businesses occupy a crucial place in the country’s development. The production increases national income, generates job opportunities, and enhances the balance of payments status, not only by generating exportable goods and by replacing imports, but also by promoting and stimulating growth in other economic sectors [15]. In recent years the industrial sector of Pakistan has achieved encouraging and broad-based growth [16]. The large-scale factories are located in urban areas while the SMEs are located in small towns and rural areas; such units of SMEs in rural areas are of great value for providing jobs to the poor rural workers [17]. To facilitate the SME sector as a development trigger, it is most important to provide adequate access to credit and other financial sources [18]. Rural finance is considered to be a vital instrument in rural development and poverty reduction. There are several tools aimed at the provision of access to credit to rural businesses and SMEs, from the provision of favorable credit lines through commercial banks to fostering the development of credit cooperation [19].

Keeping focused on the above situation, and despite all these significances and vital contributions to the economy of the country, still, the SME sector in Pakistan has many constrain, for instance, infrastructure, lack of finance, tax rate, and high-interest rate, etc. According to a report of the Multidimensional Poverty Index, Khyber Pakhtunkhwa (KPK) is the second-highest poor province of Pakistan. The proportion of people identified as multidimensionally poor in urban areas is significantly lower than in rural areas– 10.2 percent and 57.8 percent respectively. Also, in KPK poverty level is decreasing at a very slow rate. Therefore, in this area, our study observed how the SME sector supported the rural inhabitants in fighting poverty. A considerable number of studies have been carried out in the past literature regarding SMEs and rural development [11], however, there is no study with the mediation effect of “access of SMEs to finance” between the link of SME’s extension and rural development. This paper aims to define the role of SMEs in rural regions of Pakistan, (which are largely influenced by agriculture). Furthermore, this paper deals with the mediator’s influence of “access of SMEs to finance” between the links of SMEs and rural development. Hence, our study is built on a new idea that aims to notice the supposed association.

The rest of the article consists of the subsequent sections. Second, relevant literature is reviewed and hypotheses are developed. In Section three, study methods are presented. In Section Four, the results of the study are presented. Furthermore, Section five includes a discussion, the limitations of the study and future research directions are discussed.

## Theories and hypotheses

Rural areas are defined by Abban [20], are areas with some or all of the following features: (a) an area of inhabitants where most of the people are engaged in primary economic activities such as food cropping, subsistence animal husbandry, fishing, petty trading, hunting, etc. (b) a location of the country where the per-capita income is considerably lower than the national average; and (c) location of the country where the population lacks basic social facilities such as potable drinking water, health and sanitation facilities, electricity, motorable roads, and recreational facilities.

Rural development is generally denoted as the initiatives actions and movements taken to improve the standard of living in rural areas, non-urban neighborhoods, remote villages, and countryside. These communities are characterized by a low-ratio of inhabitants to open space. In this scenario, agricultural activities may be prominent while economic activities would apply to the primary sector, food processing, and raw materials [21]. Rural development aims to find ways of improving rural life with rural people’s participation to meet the needs of rural areas. The outsiders cannot understand the dominant local area environment, culture, language, and other issues. As such, local people have to engage themselves in their sustainable rural development [22].

The integrated approaches to development are being taken up in developing countries. In the next context, many ideas and approaches have been developed and followed up, for instance, the Association of Southeast Asian Nations (ASEAN) started a coordinated plan of Action on Rural Development and Poverty Eradication, others like Rapid Rural Appraisal (RRA) and Participatory Rural Appraisal (PRA), etc.

**Fig 1:**
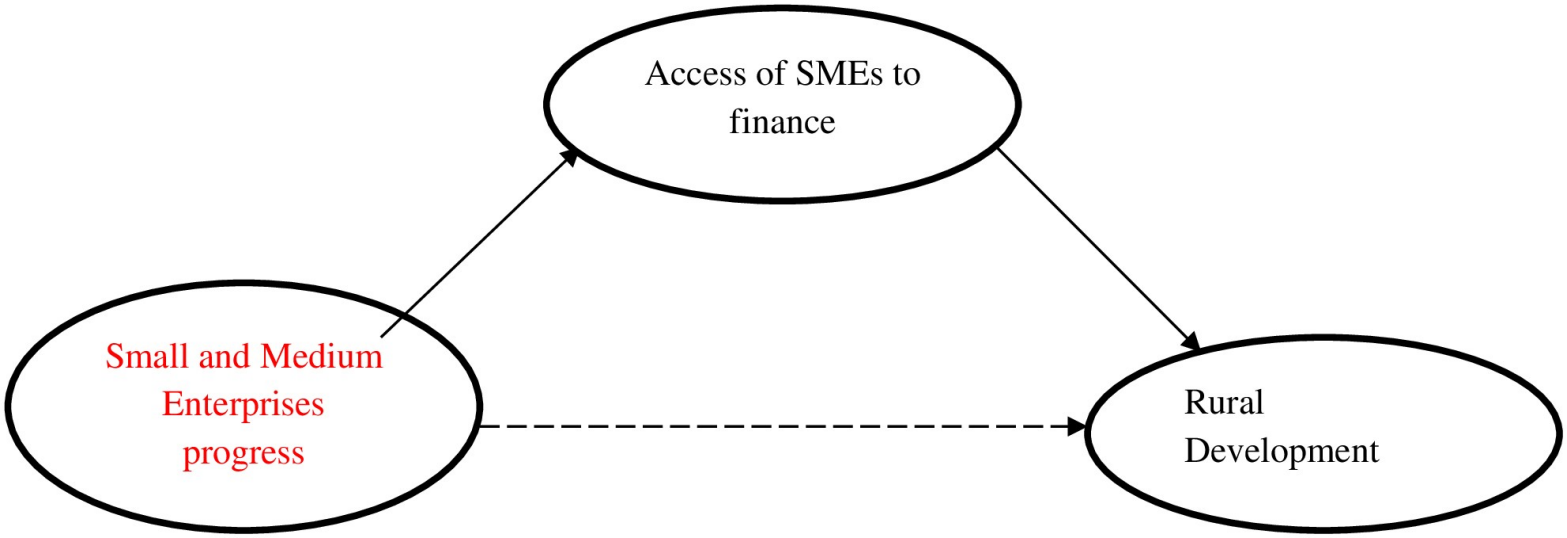
The hypothesized model of the study.

Literature showed that numerous research studies have been developed previously regarding the role of SMEs in rural development in developing countries [23, 24] as well as developed countries [25, 26]. For instance, Straka and Stávková [27], have conducted their research about the impact of SMEs on standards of living in Czech rural households. According to them, there is a strong correlation between SMEs and rural development. The findings of their study indicated the high satisfaction of the rural household with their standard of living. Reflecting on the previous studies the hypothesized model of this study is exposed in Fig 1, where the direct and indirect association between studied variables is shown. In developing countries, like Pakistan studies about SMEs and rural development is very limited. Also, previous studies have shown a direct association between SMEs and rural development [28], SMEs and economic growth [29, 30], as well as SMEs and poverty alleviation [5, 31].

Furthermore, access to finance has a strong role in SME’s establishment in the country. This sector is a vehicle for providing jobs and helping to reduce poverty [32]. In the initial stage of enterprises, entrepreneurs need a huge capital amount for the advancement of their firms [33]. Particularly in their early years, makes it difficult for investors to evaluate their risk and profit. Ikasari, Sumransat [34] indicated that SMEs in Indonesia and Thailand are perceived to have good access to finance, and have a positive impact on the establishment of enterprises in rural areas, alternatively accommodating rural development. Moreover, Deakins, Whittam [35] demonstrated that access to bank credit, loans, and funding is validated for real entrepreneur business proposals in Scotland; which is very effective for SME’s launch. Likewise, according to Quartey, Turkson [36] SMEs in Africa have inadequate access to credit and business finance, which therefore hampers their emergence and eventual development.

In developing countries, “SME’s access to finance” has many challenges that have caused the death of many firms at the beginning [37]. SMEs have positively contributed to economic development and generating employment in the least-developed nations, despite this the failure rate of SMEs is very high in these nations. One of the factors limiting the survival and growth of SMEs in these countries is the non-availability of finance [38]. Commercial banks, micro-credit institutions, community groups, business development, and business finance programs must work together to remove barriers to SME access to finance.

The above argument has shown that SME’s access to finance plays an important role in the development of SMEs, as well in the rural areas this sector can boost the living standards of rural inhabitants. This sector can cause rural development in rural regions of developing countries.

Therefore, it is plausible, based on the aforementioned claim, that SMEs play a vital role in rural development. Hence, we posit that:

**Hypothesis 1 (H1)**: *SME’s progress has a positive relationship with rural development*.
**Hypothesis 2 (H2)**: *SME’s expansion has a positive correlation with access to finance*.
**Hypothesis 3 (H3)**: “*Access of SMEs to finance” has a positive correlation with rural development*.

Previous academic debate shows that SMEs have a positive and significant contribution to rural development [13], easy access of SMEs to finance is beneficial for the improvement of this sector in rural areas [34]. According to our knowledge, no research study has been analyzed the mediating role of “access of SMEs to finance” on the association of SME’s evolution and rural development. Hence, this study is based on a novel idea that aims to explore the relationship of SMEs and rural development with the mechanism of “access of SMEs to finance”. Therefore we assume the following hypothesis that:

**Hypothesis 4 (H4)**: *“Access of SMEs to finance” mediates the relationship between SME’s progress and rural development*.

## Materials and methods

### Study site and data collection technique

This study is conducted in rural areas of Khyber Pakhtunkhwa (abbreviated by KPK) province, Pakistan. Numerous attempts have been made to secure a database of rural SMEs in research areas but to no avail.

**Fig 2:**
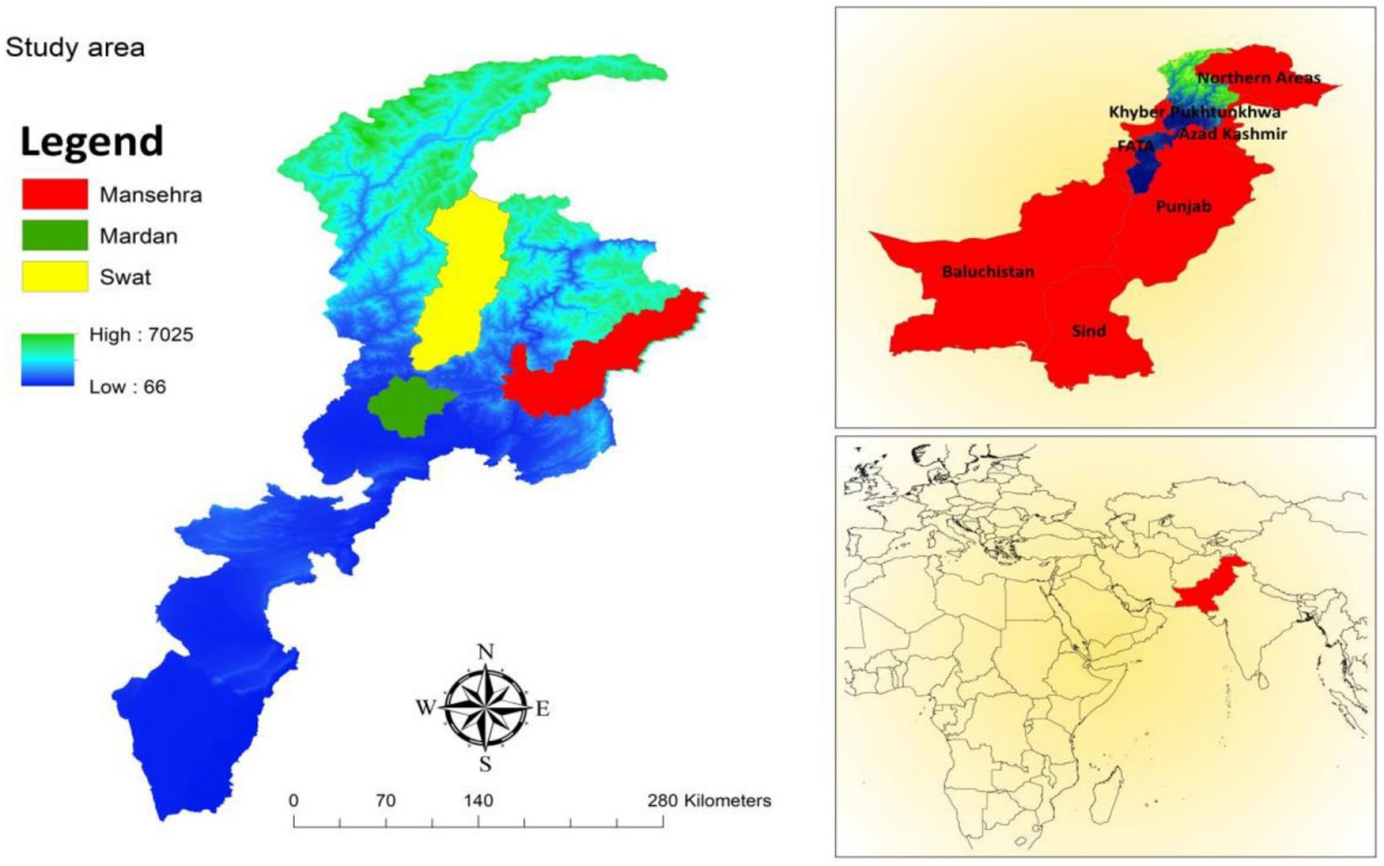
Map of the study area (ArcGIS 10.3).

**Fig 3:**
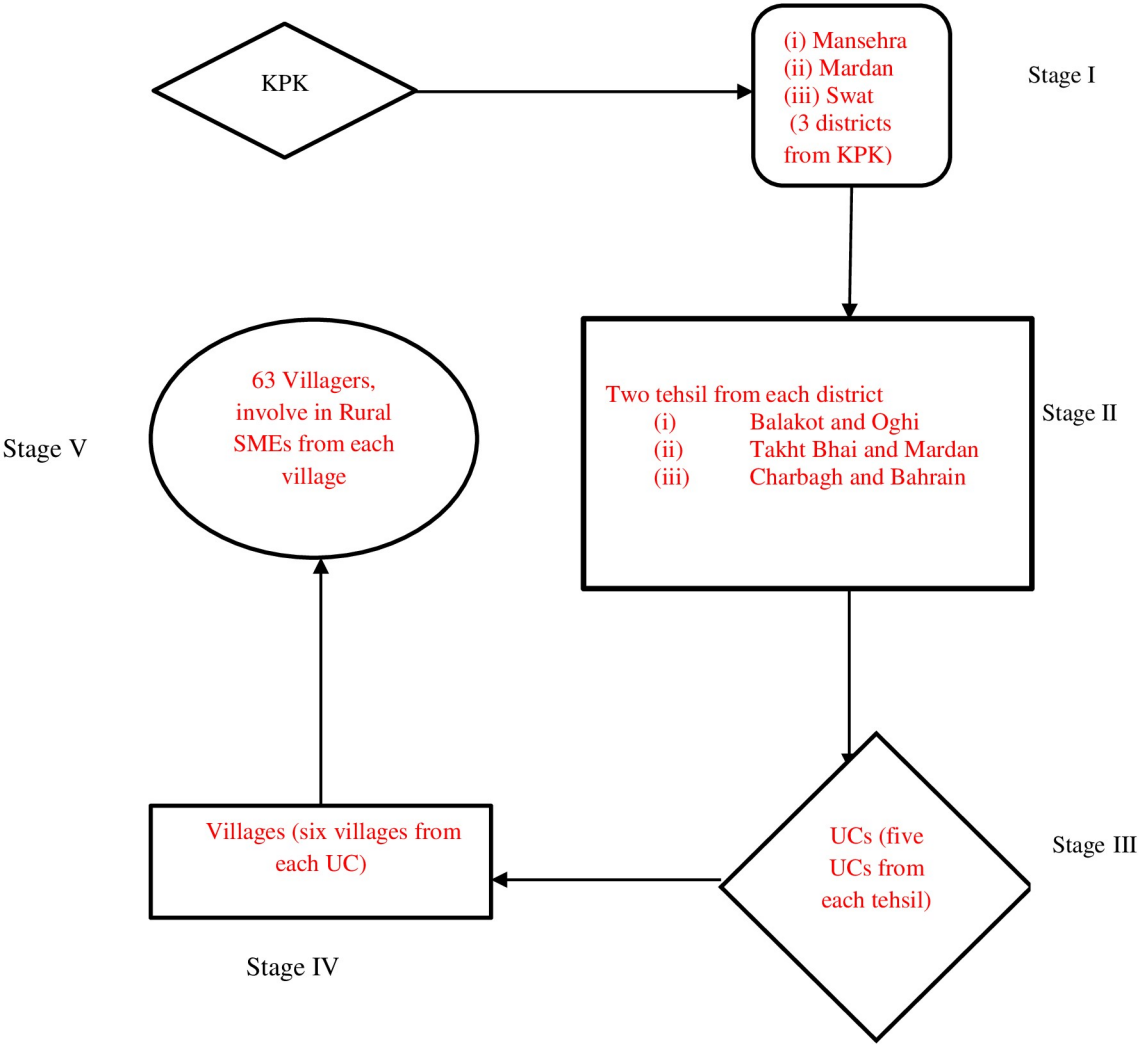
Stages of sampling to select sample.

We selected KPK as the main study area, according to our knowledge in KPK rural SMEs are more rather than other provinces. We have selected three districts namely Mansehra, Mardan, and Swat which are shown in Fig 2. The KPK province consists of 26 districts and under the KPK Local Government Act, 2013 that the province was divided into 2,989 villages, and 504 neighborhood councils. The village councils are formed in the rural regions; whereas neighborhood councils comprise urban areas. The sample universe includes rural entrepreneurs, the inhabitants of the selected study area who were actively and directly involved in SMEs in rural areas of KPK. A multi-stage stratified random sampling technique (Fig 3) was used to select sites for the study [39].

In the first stage, for the selection of study regions are three districts of KPK, keeping in view that these districts have many SMEs. In the second stage, two tehsils were randomly selected from each district. One district is administratively subdivided into parts that are called Tehsil. In the third stage, we have selected five Union Councils (UCs) from each tehsil by using stratified sampling. Here, a UC refers to a sub-section of the city administrative government (tehsil) in Pakistan. One UC may consist of several villages [40]. We skipped the urban UCs. In the fourth stage, six villages are randomly selected from each UC by using Pakistan’s village statistics [41]. In the fifth and last stage, sixty-three rural SME entrepreneurs’ are randomly selected from each village.

This study used primary data through a survey, which was obtained mainly with the instruments of self-administrated questionnaires method. Study participants were SMEs entrepreneurs (in rural areas) and the design of the questionnaire was simple and respondent-friendly. These questionnaires were initially developed in English after that transformed them into Urdu (local language) for the well understanding of the local rural entrepreneurs. This survey was conducted from late February to April 2019. We got back 338 final responses truly filled from the 380 respondents, originally 380 questionnaires were distributed among SME entrepreneurs (selection of SME’s entrepreneurs was according to Fig 3).

### Measurement of variables

For this study, three main variables are used i.e. SME’s evolution, access of SMEs to finance, and rural development. In the study, the explanatory variable is SME’s progress or evolution which is based on SME’s annual growth, existence profit as compare to previous years, evolution, profit margin, and risk base. We adopted the SME scale with 9 items from the doctoral dissertation of Vijayakumar [42]. An example question for SMEs is: “I am satisfied with business profit annually” with an alpha reliability of 0.910. For the “access of SMEs to finance” 11 items scale is used, which is taken from the study of Ikasari, Sumransat [34]. A sample item for the “access of SMEs to finance” is “the loan interest is affordable” with Cronbach’s alpha of 0.940. This study used 12 questions for measuring rural development developed by Vijayakumar [42]. In this study, we measure “rural development” as outcome variables which are measured through a proxy of economic condition and quality of life/living standard of rural entrepreneurs, and the example question is “The quality of my life improved as a result of this business” with α reliability of 0.954. Moreover, a five-point Likert scale [43], which ranges from 5 = “strongly agree” and 1 = “strongly disagree” used to reflect the agreement of the respondents of the study.

Furthermore, to check for common method bias (CMB), a post-hoc test was applied using Harman’s one-factor [44]. In this, the first factor accounted for 36.41%, which is less than the critical 50%. Therefore, no main signs of CMB were noted [45].

### Demographic characteristics

This study contains some demographic characteristic which holds a profile of entrepreneurs and profile of enterprise, clearly presented in Table 1. Profile of entrepreneurs includes gender, age, education, length of time an entrepreneur has been in business and, a region where they belong. The majority of respondents were men (55.6%), and most entrepreneurs were in the age group of 40–49 years. Almost, 39.6% was the high rate of primary level education of respondents, and approximately, most of them have 10–14 years’ experience with their business (34.9%). Regarding the participant’s regions, a large number belonged to the Mansehra district. The number of respondents from the Mardan district is very low because entrepreneurs from the Mardan district were not willing to participate in the study. Furthermore, age gender, and education are employed as control variables in the study.

**Table 1:** Demographic characteristic.

Profile of Entrepreneur	Number (n)	Percentage
Gender		
Male	188	55.6
Female	150	44.4
**Total**	338	100
Age		
Below 29	56	16.6
30–39	25	7.4
40–49	128	37.9
50–60	90	26.6
Above 60	39	11.5
**Total**	338	100
Qualification		
Illiterate	80	23.7
Primary	134	39.6
Matric	86	25.4
Above Matric	38	11.2
**Total**	338	99.9
Length of Time		
01–04 years	70	20.7
05–09 years	53	15.7
10–14 years	118	34.9
15–19 years	48	14.2
More than 20 Years	49	14.5
**Total**	338	100
Region		
Mansehra	147	43.5
Mardan	66	19.5
Swat	125	37
**Total**	338	100
**Profile of Enterprise**		
Forms of Ownership		
Sole Proprietor	240	71
Partnership	98	29
**Total**	338	100
Nature of Business		
Small	293	86.7
Medium	45	13.3
**Total**	338	100
Type of Enterprise		
Mining and Quarrying	12	3.6
Food, Beverages, and Tobacco	50	14.8
Textile and Leather	77	22.8
Wood and Wood products	36	10.7
Paper and Paper products	23	6.8
Grain milling	62	18.3
Dairy, poultry, and fisheries	53	15.7
Basic Metal products	25	7.4
**Total**	338	100

Same, Table 1 profile of enterprises showed that the majority respondent’s form of ownership was sole proprietor (71%), nature of the business was small (86.7%) and about the type of enterprise, 22.8% of the rural business were textile and leather.

### Ethics statement

The study involving human participants were reviewed and approved by the Research Ethical Committee of Zhejiang University, Hangzhou, China. The participants provided their written informed consent to participate in this study.

## Results

For data analysis SPSS (v.25) and AMOS (v.23) were used as a statistical tool, and to test the hypotheses, we applied structural equation modeling (SEM) techniques.

### Descriptive statistics

Table 2 demonstrated the descriptive statistics, correlations between the variables, and average variance extracted (AVE). Table 2 reveals the significant correlation between SMEs (firm’s growth or expansion) and “access of SMEs to finance” (r = 0.411, p< 0.01), SMEs and rural development (r = 0.434, p< 0.01) and “access of SMEs to finance” to rural development (r = 0.257, p< 0.01). The mean and standard deviation of the study variables are depicted in the table, the value of AVE meets the 0.5 minimum value for convergent validity [46, 47]. In the same table, diagonal values reveal the discriminant validity, Table 2 displays that AVE’s square root of all variables exceeds all of the other associations involving that construct [48, 49].

**Table 2:** Descriptive statistics and correlation.

n = 338	Mean	St. div	AVE	Correlation		
				1	2	3
1. SME	3.9816	0.65533	0.532	**0.729**		
2. ASF	3.8956	0.76633	0.592	.411**	**0.770**	
3. RD	3.8575	0.83050	0.635	.434**	.257**	**0.797**

Note: Correlation is significant at the 0.01 level (2-tailed).

SME: Small and Medium Enterprises

ASF: Access of SMEs to Finance

RD: Rural Development

AVE: Average variance extracted

Bold values are the square root of AVE revealing discriminant validity.

### Measurement model

We developed a measurement model based on our study variables, Cronbach’s Alpha, standardized factor loading, and composite reliability (C.R) are explained in Table 3. The Alpha value of entire variables is greater than the acceptance criteria that is 0.70 [46, 50]. Likewise, standardized factor loading values are also shown in the same table and values met the threshold criteria, which are ranged from 0.53 to 0.85. as per the recommendation of Hair [51], factor loadings above 0.5 are considered significant, hence the loadings providing a significant contribution for each construct. Also, the value of composite reliability is higher than the cutoff at 0.60 [52, 53].

**Table 3:** Factor loading of indicators and reliability.

Factors	No. of Items	Items	Cronbach Alpha	Factor loading	CFA	Composite Reliability
Small Medium Enterprises	9	SME1	0.91	0.792	0.83	0.911
		SME2		0.729	0.713	
		SME3		0.764	0.74	
		SME4		0.762	0.817	
		SME5		0.604	0.615	
		SME6		0.657	0.681	
		SME7		0.671	0.642	
		SME8		0.786	0.768	
		SME9		0.723	0.728	
Access of SMEs to Finance	11	ASF1	0.94	0.825	0.844	0.93
		ASF2		0.827	0.812	
		ASF3		0.828	0.823	
		ASF4		0.837	0.837	
		ASF5		0.723	0.703	
		ASF6		0.760	0.756	
		ASF7		0.831	0.805	
		ASF8		0.825	0.828	
		ASF9		0.807	0.807	
		ASF10		0.647	0.655	
		ASF11		0.529	0.539	
Rural Development	12	RD1	0.954	0.809	0.831	0.956
		RD2		0.816	0.812	
		RD3		0.797	0.802	
		RD4		0.808	0.835	
		RD5		0.742	0.735	
		RD6		0.773	0.756	
		RD7		0.841	0.815	
		RD8		0.831	0.836	
		RD9		0.737	0.755	
		RD10		0.780	0.774	
		RD11		0.833	0.842	
		RD12		0.778	0.757	

### Confirmatory factor analysis

An exploratory factor analysis (EFA) was performed on the sample to identify any abnormal items. The results showed that all the variables load on the expected factors, thus indicating that there was nothing wrong with the data. We then based on EFA continued to test the confirmatory factor analysis (CFA) model with the study sample. Full information maximum likelihood (FIML) estimation as suggested by Schafer and Graham [54] and Asif, Jameel [55] to deal with missing data, but our data was normal. The results of the CFA model were acceptable, which are presented in Table 4.

**Table 4:** Model fit statistics.

Fit Indices	Estimated Value	Threshold	Comments
Chi-square	978.95		
Degrees of freedom	459		
CMIN/DF	2.133	Between 1 and 3	Excellent
CFI	0.933	>0.95	Acceptable
IFI	0.934	>0.90	Excellent
TLI	0.928	>0.90	Excellent
SRMR	0.049	<0.08	Excellent
RMSEA	0.058	<0.06	Excellent

Note: DF: Degree of Freedom; CFI: Comparative Fit Index;

IFI: Incremental Fit Measures; TLI: Tucker-Lewis Index;

SRMR: Standardized Root Mean Square Residual;

RMSEA: Root Mean Square Error of Approximation

CFA is a commonly applied approach and is used before performing mediation analysis [56]. Table 4 demonstrated that value of CFA model is (Chi-square = 978.95, df = 459, CFI = 0.933, IFI = 0.934, TLI = 0.928, SRMR = 0.049, RMSEA = 0.058). The suggested acceptance of a good fit to a model requires that the value of Comparative Fit Index (CFI) and Tucker-Lewis Index (TLI) be greater than or equal to 0.90 [57] but according to West, Taylor [58] TLI and CFI standard values be greater than 0.95 [58].

The root mean square error of approximation (RMSEA) and standardized root mean square residual (SRMR) met the required acceptable range of less than 0.06 and 0.08 respectively [46, 59]. For the whole sample, the overall goodness-of-fit indices revealed that the model had a satisfactory fit with the data. Measurement model Table 5 added validate construct validity as mentioned by Barroso Castro, Villegas Perinan [60], and Qing, Asif [61].

**Table 5:** Measurement model for a) explanatory (SMEs) and b) outcome variable (rural development) and c) mediator variable (access of SMEs to Finance).

Indicators	Small and Medium Enterprises			Access of SMEs to Finance			Rural Development		
	Standardized regression weights	T	R^2^	Standardized regression weights	T	R^2^	Standardized regression weights	T	R^2^
SME1	0.830	Fixed	0.688						
SME2	0.713	14.593	0.508						
SME3	0.740	15.348	0.547						
SME4	0.817	17.675	0.667						
SME5	0.615	12.053	0.378						
SME6	0.681	13.732	0.463						
SME7	0.642	12.727	0.412						
SME8	0.768	16.159	0.589						
SME9	0.728	15.019	0.529						
ASF1				0.844	Fixed	0.712			
ASF2				0.812	18.256	0.659			
ASF3				0.823	18.629	0.677			
ASF4				0.837	19.402	0.700			
ASF5				0.703	14.771	0.494			
ASF6				0.756	16.363	0.571			
ASF7				0.805	17.983	0.648			
ASF8				0.828	18.810	0.685			
ASF9				0.807	18.058	0.651			
ASF10				0.655	13.414	0.429			
ASF11				0.539	10.530	0.290			
RD1							0.831	Fixed	0.690
RD2							0.812	17.648	0.659
RD3							0.802	17.294	0.643
RD4							0.835	18.375	0.697
RD5							0.735	15.339	0.540
RD6							0.756	18.883	0.571
RD7							0.815	18.253	0.664
RD8							0.836	18.406	0.698
RD9							0.755	15.899	0.570
RD10							0.774	16.431	0.553
RD11							0.842	18.622	0.708
RD12							0.757	15.952	0.573

Note: SME; Small & Medium Enterprises, ASF; Access of SMEs to Finance, RD; Rural Development

### Hypotheses testing & structural equation model

For testing the hypotheses 1 to 3, we applied structural equation modeling and estimated the coefficients (β) with a 95 percent bootstrapping confidence interval. Table 6 summarizes the hypotheses testing in this study; which is presented as the results of coefficients (β) and the p-value. Since hypothesis 1 predicted there will be a significant and positive relationship between SME’s progress and rural development As a confirmation from Table 6, we found support for hypothesis 1 (β = 0.435; CR 6.693, P < 0.01). Hypothesis 2 predicted there will be a significant and positive relationship between SMEs progress and “access of SMEs to finance”. We found support for hypothesis 2 (β = 0.481; CR = 8.274, P< 0.01). Likewise, Hypothesis 3 predicted there will be a significant and positive relationship between “access of SMEs to finance” and rural development. As evidenced from Table 6 we found support for hypothesis 3 (β = 0.176; CR = 3.164; P < 0.05).

**Table 6:** Regression coefficients for testing hypotheses 1–3.

Hypotheses	Path	B	S.E	C.R	Sig.
H1	SMEs → RD	0.435	0.65	6.693	<0.01 (***)
H2	SMEs → ASF	0.481	0.58	8.274	<0.01 (***)
H3	ASF → RD	0.176	0.56	3.164	<0.05 (**)

Note: SME: Small and Medium Enterprises growth

ASF: Access of SMEs to Finance

RD: Rural Development

**Fig 4:**
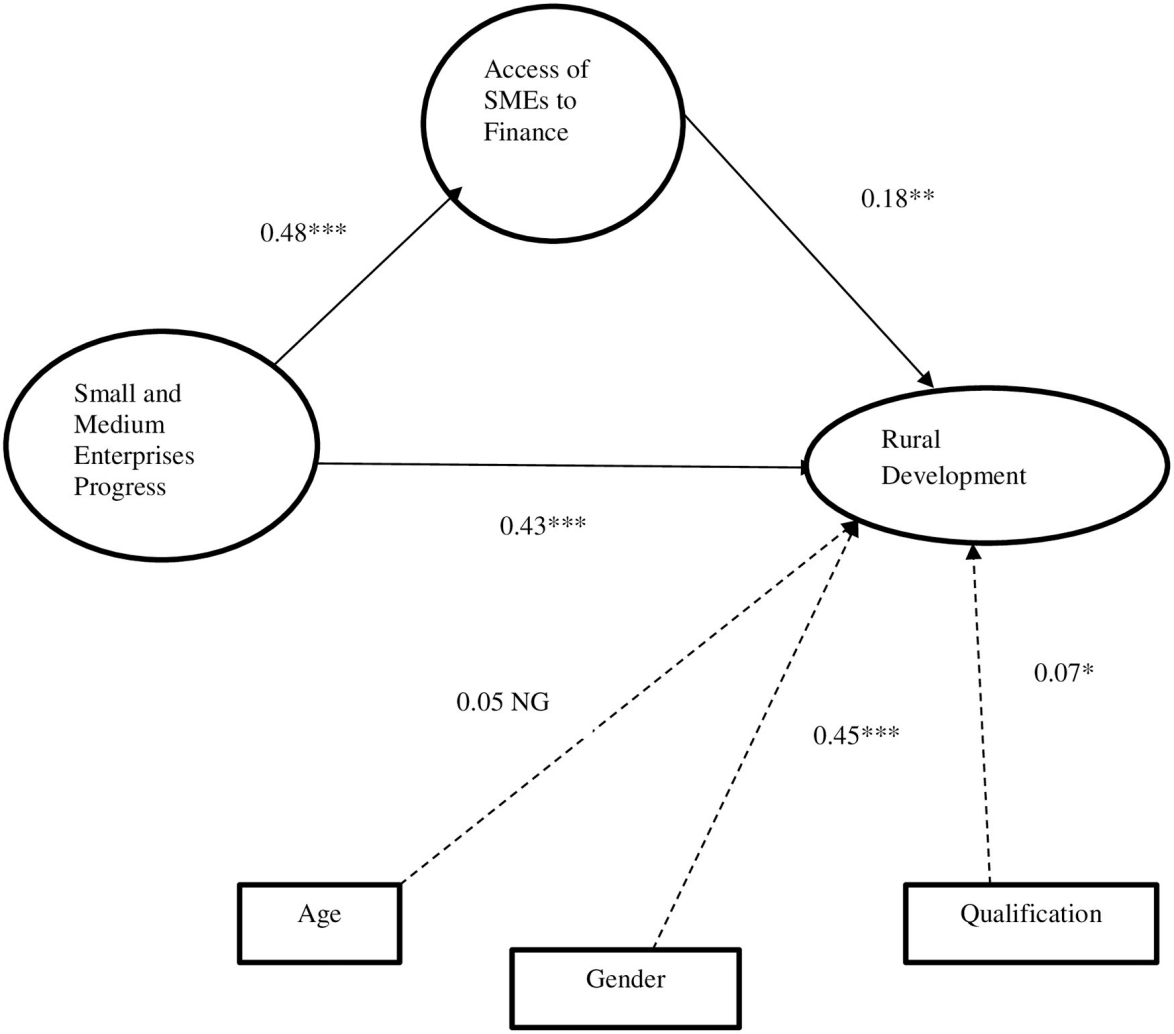
SEM results for mediation. *** p < 0.01, ** p < 0.05, * p < 0.1, NS; Non Significant.

Turning to the fourth hypothesis, which expected that “access of SMEs to finance” mediates the relationship between SMEs and rural development. Fig 4 showed the result of the mediating relationship of H4. “Access of SMEs to finance” was added to the model as a mediating variable. Fig 4 uncovers the mediating role of “access of SMEs to finance” in the link between SMEs and rural development. As demonstrated Fig 4 the path value from SMEs to access of SMEs to finance (β = 0.48; p< 0.01), from access of SMEs to finance to rural development (β = 0.18; p< 0.05) and SMEs to rural development (β = 0.43; p< 0.01). These findings revealed that “access of SMEs to finance” partially mediates the link between SMEs and rural development.

In line with the above evidence, we performed percentile bootstrapping and bias-corrected bootstrapping at 95% confidence interval with 5000 bootstrap sample [62] to exam complete or partial mediation. We performed the confidence of interval of the lower and upper bounds to test the significance of indirect effects as suggested by Preacher and Hayes [63]. As seen in Table 7, we found that the indirect effects of “access of SMEs to finance” on rural development (standardized β = 0.066, p < 0.05) are significant. The direct relationship between SME’s progress and rural development (β = 0.345, p = < 0.01) is significant and supported Hypothesis 4 with partial mediation. Therefore, evidence of the partial mediation mechanism proved H4. For the full mediation, the direct relationship between the independent variable (SMEs) and the outcome variable (RD) must be insignificant [64, 65]. It is clear from Fig 4 that the direct path from SMEs to RD is significant in the presence of a mediator and provides support for partial mediation.

**Table 7:** Results of bootstrapping for standardized direct, indirect, and total effects of the model.

	Coeff.	Std. E	Bootstrapping				Sig.
			Bias-corrected percentile 95%		Percentile method 95%		
			LLCI	ULCI	LLCI	ULCI	
**Standardized direct effects**							
SME → RD	0.342	0.083	0.179	0.503	0.179	0.502	***
ASF → RD	0.162	0.069	0.032	0.301	0.022	0.295	***
SME → ASF	0.411	0.074	0.259	0.550	0.255	0.547	**
**Standardized indirect effects**							
SME → ASF → RD	0.066	0.031	0.017	0.149	0.009	0.136	**
**Standardized total effects**							
SME → RD	0.408	0.076	0.259	0.551	0.253	0.547	***
ASF → RD	0.162	0.069	0.032	0.301	0.022	0.295	***
SME → ASF	0.411	0.074	0.259	0.550	0.255	0.547	**

Note: LLCI = lower level of confidence interval; ULCI upper level of confidence interval. Sig:

**p<0.05,

***p = and <0.01

## Discussion

The main purpose of the present study is to test the influence of SME’s growth and development on rural development, through the intervening role of access of SMEs to finance. The findings of this study showed a significant association of SME’s expansion, access of SMEs to finance, and rural development, while the access of SMEs to finance mediates the association among SMEs and rural development. SMEs are a driving force for developing the economy, creating employment, and basic tools for alleviating poverty. This means that SMEs can accommodate rural entrepreneurs regarding their standard of living and improving their quality of life. These results are in line with numerous previous studies of Oboniye [66], Xiujuan [67], Vasilescu and Popa [68], and Tambunan [69]. For instance, Xiujuan [67] claimed that SME’s development in rural areas is conducive to promoting rural economic growth, and SMEs have become the bottleneck of rural development. Furthermore, Vasilescu and Popa [68] stated that the SME sector is the key to “the triple” of the rural durable development: economic, social, environment. Tambunan [69] explained that clustering for rural SMEs development is development in rural areas in Indonesia.

Nevertheless, SME sector expansion was found to hold significant influence by the access of SMEs to finance. Access of SMEs to finance is most beneficial for developing this sector to a large extent. These findings are in line with the findings of Ikasari, Sumransat [34], Saleem [70], and Abor and Quartey [71]. They suggested that SME’s development is possible in rural areas due to feasible and viable accessibility of finance and loan. They also recommended that constraints in finance should be overcome in the least- developed countries for good development in SMEs in the rural region.

Furthermore, as for the relationship between access of SMEs to finance and rural development, our study found that “access of SMEs to finance” significantly predicted rural development. When SMEs in rural areas have viable access to finance, simply means that rural SMEs entrepreneurs approach to finance for developing their rural enterprises into the larger extent, after this step they can generally more by this sector and improve their life standard, alternatively, in turn, this enhancing rural development by the access of SMEs to finance [72]. These findings are inconsistent with the previous studies findings of Lekhanya and Mason [73], and Ogujiuba, Jumare [74]. The authors argued that rural financing institutions can play major and significant roles in the development of the rural region in form of overcoming the constraints and barriers (interest rate so on.) for providing loans to rural entrepreneurs.

Additionally, the findings of this study found that in a mediation mechanism “access of SMEs to finance” significantly mediates the link between SME’s development and rural development. Further, this study exposed that “access of SMEs to finance” has a partial mediation effect on SMEs and rural development. Therefore, testing the above association through a mediator is a relatively novel idea.

This study significantly contributes to the existing literature on SME’s development and rural development by examining the unexplored side of SME’s development and rural development associations in various ways. First, previous research studies discovered that SMEs have significantly played a role in the economy of developed as well as developing countries [75, 76], hence SME’s role in alleviating poverty [31]; therefore we chose SMEs impact on rural development in one of the developing country, Pakistan. Second, many studies explored the role of SMEs in economic growth, poverty reduction, approximately in every nation. But no study has employed “access of SMEs to finance” as a mediator. Third, very scarce research studies have been conducted to investigate rural SMEs and rural development in the Pakistani context [11, 23]; but no study has been investigated by using a mediating mechanism. Hence, this is the first research to investigate the impact of rural SMEs on rural development through “access of SMEs to finance” in the rural areas of KPK, Pakistan.

### Implications, limitation and future directions

The findings of this research study hold essential implications for both theoretically and practically. Theoretically, unlike many prior research studies, not all rural SME development patterns and methods were considered relevant in the Pakistan context. Though we debate that such outcomes and findings are due to development factors and rural specificities related to the developing country’s setting (KPK, Pakistan). We encourage researchers and practitioners for further research to discover with more depth the intervening influence of “other determinants/factors for development” in the link between SME’s expansion and rural development. Furthermore, that inadequate and inconclusive outcomes thus far about the various rural SMEs of different rural regions in the country on rural development could be described by the intervening role of “access of SMEs to finance” in such a process. In this vein, we have revealed that “access of SMEs to finance” does mediate the relationship between SMEs and rural development. In other words, the progress in the SME sector could be subject to rural development. Therefore, a future research study is also needed to clarify this role.

With the practical perspective, the findings of this study hold some vital implications to policymakers, loan institutions, and departments for SMEs in Pakistan particularly and in developing countries in general. The SME sector is found to be a good predecessor to rural development, which would in turn improve the living standard of residents and improve their quality of life. Therefore, credit-issuing institutions, departments, and commercial banks, which provide loan for the establishment of SMEs; they should overcome constraints such as low-interest rate, as well as omit the favoritism and nepotism strategy.

The following should be acknowledged in terms of limitations. First, though this study is specific to one province of Pakistan, further study should be conducted in the rest of the provinces. Second, this study is in the context of Pakistan, we call for further qualitative studies to explain such in other developing countries to increase the results generalizability. Third, this study used survey data, which is collected from the rural SME’s entrepreneurs, for the cross-checking of findings future research study can be conducted through secondary data. Finally, although we controlled for several entrepreneurs’ demographics, it could be argued that such factors can mediate and moderate the connections between SME’s growth and rural development. Therefore, we also call for further studies to examine such influences.

## Supporting information

S1 Data (SAV)
